# Aryl Hydrocarbon Receptor Activation and Tissue Factor Induction by Fluid Shear Stress and Indoxyl Sulfate in Endothelial Cells

**DOI:** 10.3390/ijms21072392

**Published:** 2020-03-31

**Authors:** Guillaume Lano, Manon Laforêt, Clarissa Von Kotze, Justine Perrin, Tawfik Addi, Philippe Brunet, Stéphane Poitevin, Stéphane Burtey, Laetitia Dou

**Affiliations:** 1 Aix Marseille Univ, INSERM, INRAE, C2VN, 13005 Marseille, France; guillaume.lano@ap-hm.fr (G.L.); manon.laforet@laposte.net (M.L.); tawfik.addi@gmail.com (T.A.); philippe.brunet@ap-hm.fr (P.B.); stephane.poitevin@univ-amu.fr (S.P.); stephane.burtey@univ-amu.fr (S.B.); 2 Centre de Néphrologie et Transplantation Rénale, AP-HM, Hôpital de la Conception, 13005 Marseille, France; clarissa.von-kotze@ap-hm.fr; 3 Hôpital Sainte Musse, 83056 Toulon, France; justine.perrin@ch-toulon.fr; 4 Département de Biologie, Université d’Oran 1 Ahmed Benbella, LPNSA, 31000 Oran, Algerie

**Keywords:** aryl hydrocarbon receptor, indoxyl sulfate, tissue factor, shear stress, chronic kidney disease

## Abstract

Endogenous agonists of the transcription factor aryl hydrocarbon receptor (AHR) such as the indolic uremic toxin, indoxyl sulfate (IS), accumulate in patients with chronic kidney disease. AHR activation by indolic toxins has prothrombotic effects on the endothelium, especially via tissue factor (TF) induction. In contrast, physiological AHR activation by laminar shear stress (SS) is atheroprotective. We studied the activation of AHR and the regulation of TF by IS in cultured human umbilical vein endothelial cells subjected to laminar fluid SS (5 dynes/cm2). SS and IS markedly increased the expression of AHR target genes *PTGS2* (encoding for COX2), *AHRR*, *CYP1A1*, and *CYP1B1*, as well as *F3* (encoding for TF), in an AHR-dependent way. IS amplified SS-induced TF mRNA and protein expression and upregulation of AHR target genes. Interestingly, tyrosine kinase inhibition by genistein decreased SS- but not IS-induced TF expression. Finally, the increase in TF expression induced by laminar SS was not associated with increased TF activity. In contrast, IS increased TF activity, even under antithrombotic SS conditions. In conclusion, IS and SS induce AHR activation and AHR-dependent TF upregulation by different mechanisms. Impairment of the antithrombotic properties of shear stressed endothelium by toxic AHR agonists could favor cardiovascular diseases in CKD.

## 1. Introduction

Chronic kidney disease (CKD) leads to a pathological accumulation of uremic toxins from tryptophan metabolism, such as indoxyl sulfate (IS), indole-3 acetic acid (IAA), and kynurenine [1]. These toxins are agonists of the transcription factor aryl hydrocarbon receptor (AHR) [1,2,3] and induce its activation in different cells, notably endothelial cells [1,4]. The accumulation of AHR agonists in CKD patients is deleterious [4,5], and many of the harmful effects of tryptophan-derived uremic toxins are related to their AHR-activating ability [1,4].

In the absence of ligands, AHR is retained in the cytoplasm in a complex with chaperone proteins HSP90, a co-chaperone p23, and AIP [6]. Upon ligand binding, AHR translocates to the nucleus where it forms a heterodimer with the aryl hydrocarbon nuclear translocator (ARNT), which recruits additional transcriptional cofactors [6]. The AHR/ARNT heterodimer binds to the xenobiotic response element (XRE) in the promoter region of a variety of genes called the “AHR gene battery” to modulate their transcription [6]. The cytochromes P450 CYP1A1 and CYP1B1 [7] and the AHR repressor (AHRR) [8] are regulated by this AHR genomic pathway. COX2 regulation via AHR is somewhat different, because COX2 can be regulated via both the genomic and the non-genomic pathways of AHR activation, depending on the cell type and AHR agonist [9,10,11].

While AHR can directly regulate transcription via the genomic pathway, it can also indirectly regulate gene expression by activating signaling molecules and other transcription factors in a so-called non-genomic pathway. This is the case of AHR-mediated regulation of endothelial tissue factor (TF), the principal initiator of coagulation [12]. Indeed, we reported that endothelial TF upregulation by the AHR agonist IAA is transcriptional [13]. However, unlike the regulation via the AHR genomic pathway, the AHR-mediated endothelial TF upregulation does not involve AHR binding to the promoter of *F3* gene encoding for TF. With IAA, we demonstrated that it occurs via a non-genomic pathway in which AHR activates p38 MAPK, which then induces NF-κB activation, leading to NF-κB binding to *F3* promoter [13]. 

In addition to stimulation by its ligands, AHR can be strongly activated in endothelial cells by hemodynamic forces such as fluid shear stress [14,15]. Using CYP1A1 and CYP1B1 upregulation, Conway et al. demonstrated that AHR activation depends on the shear stress magnitude and time-average [14]. Study of the mouse aorta has shown the influence of the hemodynamic environment, which induces shear stress modifications, on AHR activation including increased nuclear AHR localization and CYP1A1 expression in thoracic aorta, and reduced AHR nuclear localization and CYP1A1 expression in the region of lesser curvature [14].

Laminar shear stress is an essential element in the vascular function of blood vessels, and it is known to be atheroprotective [16]. Han et al. suggest that the activation of AHR in endothelial cells by laminar shear stress may have an important physiological role in regulating proliferation and protective response to xenobiotics, especially by mediating cell cycle arrest and sustained expression of *CYP1A1* [15]. In contrast, the activation of AHR by indolic uremic toxins is largely demonstrated to be harmful for endothelial cells [1] and related to cardiovascular diseases [5], through the induction of pro-atherogenic and prothrombotic mechanisms [4,17]. It is not known how pathological AHR activation induced by uremic toxins affects the endothelial response to shear-stress mediated physiological AHR activation. We therefore studied the activation of AHR by laminar fluid shear stress and the indolic uremic toxin, indoxyl sulfate. For that purpose, we examined the expression of genes that are differently regulated by AHR, with a focus on TF.

## 2. Results

### 2.1. Effect of Shear Stress and IS on AHR and AHRR Expression

**Figure 1:**
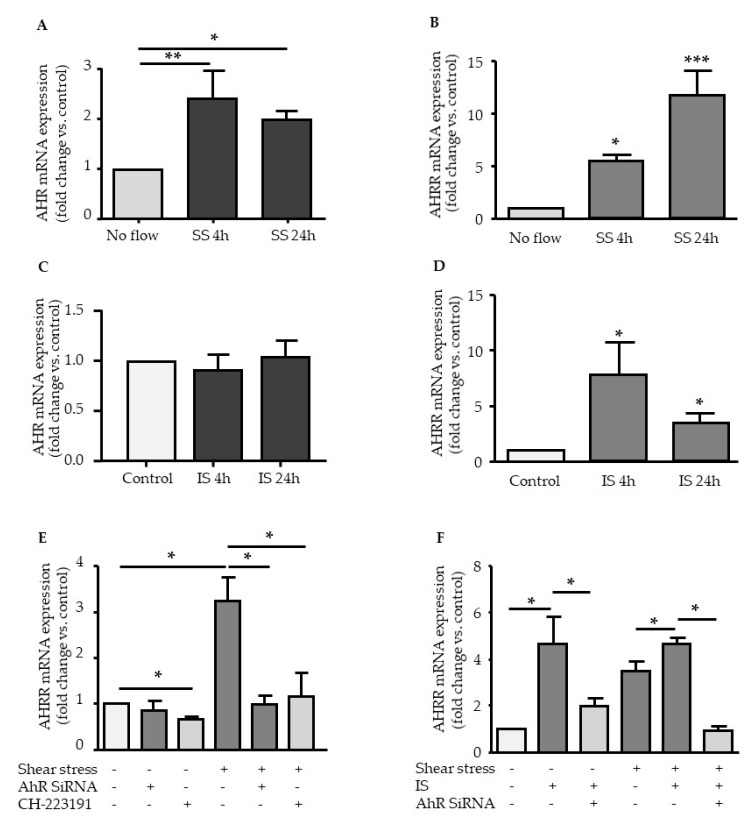
Effect of shear stress and indoxyl sulfate (IS) on aryl hydrocarbon receptor (AHR) and AHR-dependent AHR repressor (AHRR) expression. Effect of shear stress 5 dynes/cm^2^ on AHR (**A**) and AHRR (**B**) mRNA expression. Data, expressed as fold change vs. control, represent the mean ± SEM of *n* = 7 independent experiments. Effect of IS 200µM on AHR (**C**) and AHRR (**D**) mRNA expression. Data, expressed as fold change vs. control, represent the mean ± SEM of *n* = 4 independent experiments. (**E**) Effect of the AHR inhibitor CH-223191 (10µM) and of AHR siRNA on AHRR mRNA expression after 4 h of shear stress. Data represent the mean ± SEM of 5 independent experiments. (**F**) Effect of AHR siRNA on AHRR mRNA expression after a 4 h stimulation with IS 200µM. Data represent the mean ± SEM of 6 independent experiments. * *p* < 0.05, **p < 0.01, ***p < 0.001.

We first studied the mRNA expression of AHR and of its repressor AHRR in human umbilical vein endothelial cells (HUVEC) exposed for 4 h and 24 h to laminar shear stress of 5 dynes/cm2 and/or to the AHR agonist IS at 200 µM. Laminar shear stress induced sustained and increased expression of both AHR (Figure 1A) and AHRR (Figure 1B). In contrast, IS stimulation did not affect AHR expression (Figure 1C) but increased AHRR expression, which reached a maximum at 4 h, then decreased at 24 h but remained significantly high (Figure 1D). 

The role of AHR in upregulation of AHRR mediated by shear stress was studied using small interfering RNA knockdown of AHR (AHR siRNA) and the AHR inhibitor CH223191. AHR siRNA and CH223191 strongly inhibited shear stress-induced upregulation of AHRR (Figure 1E). 

When endothelial cells were stimulated with IS under shear stress conditions (Figure 1F), IS slightly amplified shear stress-induced mRNA expression of AHRR (Figure 1F). AHR inhibition by AHR siRNA significantly decreased the induction of AHRR mediated by IS under shear stress conditions, as well as under static conditions (Figure 1F). 

### 2.2. Shear Stress and IS Have AHR-Dependent Additive Effects on Upregulation of COX2, CYP1A1, and CYP1B1

**Figure 2:**
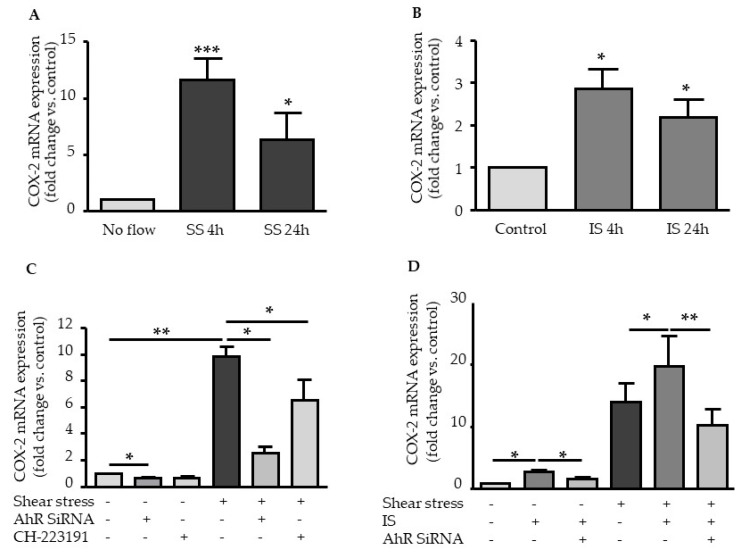
AHR-dependent additive effects of shear stress and IS on *COX2* upregulation. Effect of shear stress 5 dynes/cm^2^ (**A**) and IS 200 µM (**B**) on COX2 mRNA expression. Data, expressed as fold change vs. control, represent the mean ± SEM of *n* = 7 (A) and *n* = 4 (B) independent experiments. (**C**) Effect of the AHR inhibitor CH-223191 (10 µM) and of AHR siRNA on COX2 mRNA expression after 4 h of shear stress. Data represent the mean ± SEM of 4 independent experiments. (**D**) Effect of AHR siRNA on COX2 mRNA expression after a 4 h stimulation with IS 200µM. Data represent the mean ± SEM of 5 independent experiments. *p < 0.05, **p < 0.01, ***p < 0.001.

We then studied the upregulation of AHR target genes *PTGS2* (COX2), *CYP1A1*, and *CYP1B1* in HUVEC exposed to laminar shear stress and IS. Laminar shear stress (Figure 2A) and IS (Figure 2B) increased COX2 mRNA expression. Under shear stress conditions, COX2 induction was maximal at 4 h (COX2 mRNA fold change at 4 h: 11.6 ± 1.8) and remained sustained at 24 h (COX2 mRNA fold change at 24 h: 6.3 ± 2.3). COX2 upregulation induced by IS was lower than that induced by shear stress (COX2 mRNA fold change: 2.8 ± 0.5 at 4 h; 2.1 ± 0.4 at 24 h), whatever the time (Figure 2B). AHR siRNA and CH223191 decreased shear stress-induced upregulation of COX2 (Figure 2C). AHR siRNA suppressed COX2 upregulation by IS under static conditions (Figure 2D). When endothelial cells were stimulated with IS under shear stress, IS amplified shear stress-induced mRNA expression of COX2 (Figure 2D). AHR inhibition significantly decreased IS-induced COX2 upregulation under shear stress and static conditions (Figure 2D).

**Figure 3:**
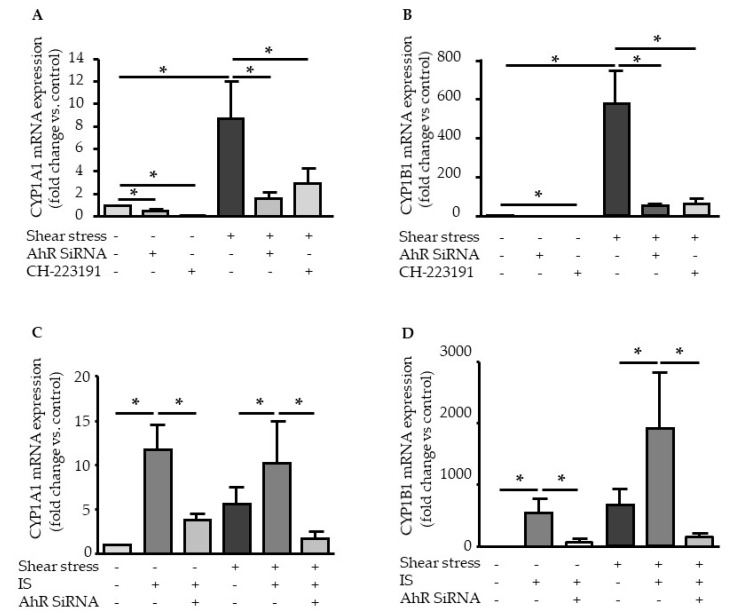
AHR-dependent additive effects of shear stress and IS on CYP1A1 and CYP1B1 upregulations. Effect of the AHR inhibitor CH-223191 (10µM) and of AHR siRNA on mRNA expression of CYP1A1 (**A**) and CYP1B1 (**B**) after 4 h of shear stress (5 dynes/cm^2^). Data represent the mean ± SEM of 5 independent experiments. Effect of AHR siRNA on mRNA expression of CYP1A1 (**C)** and CYP1B1 (**D**) after a 4 h stimulation with IS 200 µM. Data represent the mean ± SEM of 5 independent experiments. * *p* < 0.05.

We examined the involvement of AHR in the upregulation of CYP1A1 and CYP1B1 mRNA and the potential additive effects of shear stress and IS on CYP1A1 and CYP1B1 upregulation. AHR inhibition strongly decreased the upregulation of CYP1A1 and CYP1B1 induced by shear stress (Figure 3A,B) and by IS (Figure 3C,D). When endothelial cells were stimulated with IS under shear stress conditions, IS amplified shear stress-induced mRNA expressions of CYP1A1 (Figure 3C) and CYP1B1 (Figure 3D), which were significantly decreased by AHR inhibition with AHR siRNA (Figure 3C,D). 

### 2.3. Genistein Suppressed Shear Stress- and IS-Mediated AHR Target Genes Activation

**Figure 4:**
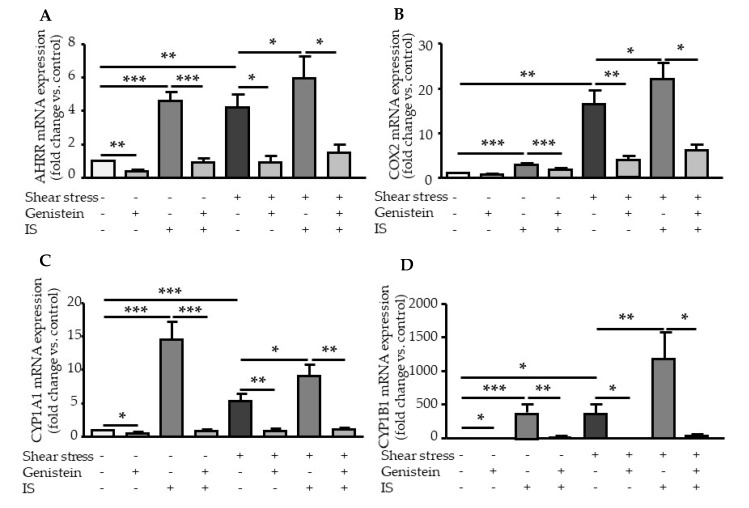
Genistein inhibits the induction of AHR target genes by shear stress and IS. Effect of genistein (100µM) on mRNA expression of AHRR (**A**), COX2 (**B**), CYP1A1 (**C**), and CYP1B1 (**D**) after 4-h of shear stress (5 dynes/cm^2^) and/or IS (200µM) stimulation. Data represent the mean ± SEM of 5 independent experiments. *p < 0.05, **p < 0.01, ***p < 0.001.

We analyzed the effect of genistein, an inhibitor of tyrosine kinases that also inhibits AHR nuclear translocation [18]. Genistein strongly inhibited the induction by shear stress of all AHR target genes: *AHRR* (Figure 4A), *PTGS2* (COX2) (Figure 4B), *CYP1A1* (Figure 4C), and *CYP1B1* (Figure 4D). In addition, genistein significantly suppressed the upregulation of AHRR (Figure 4A), COX2 (Figure 4B), CYP1A1 (Figure 4C), and CYP1B1 (Figure 4D) mRNA in HUVEC stimulated with IS under static and shear stress conditions (Figure 4A–D).

### 2.4. TF Upregulation by Shear Stress and IS is Dependent of AHR Activation

We then focused on TF (encoded by the *F3* gene) and studied its AHR-dependent upregulation in HUVEC exposed to laminar shear stress, or in cells stimulated with IS at 200 µM. 

**Figure 5:**
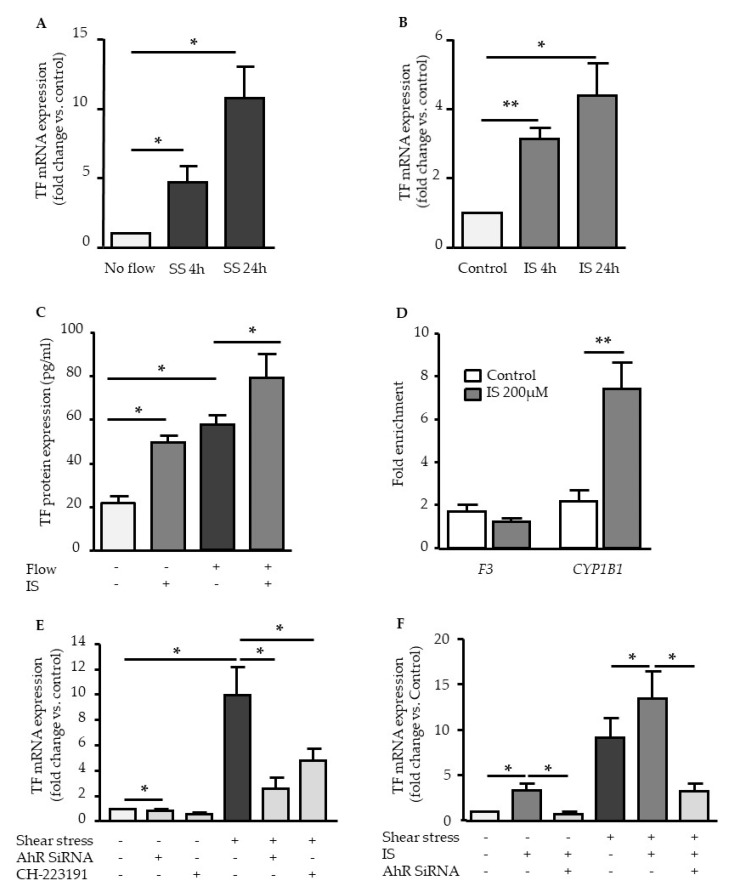
Tissue factor (TF) expression induced by shear stress and IS dependent of AHR activation. Effect of shear stress 5 dynes/cm^2^ (**A**) and IS 200µM (**B**) on TF mRNA expression. Data, expressed as fold change vs. control, represent the mean ± SEM of *n* = 6 (A) and *n* = 4 (B) independent experiments. (**C**) Effect of shear stress and IS on TF protein expression after 6h of stimulation. Data, expressed in pg/mL, represent the mean ± SEM of *n* = 6 independent experiments. (**D**) AHR binding to the promoters of *F3* and *CYP1B1* was studied by chromatin immunoprecipitation (ChIP) after 1 h of human umbilical vein endothelial cells (HUVEC) stimulation by 200µM IS. Data, expressed as fold enrichment, represent the mean ± SEM of *n* = 6 independent experiments. (**E**) Effect of the AHR inhibitor CH-223191 (10µM) and of AHR siRNA on TF mRNA expression after 4 h of shear stress. Data represent the mean ± SEM of 5 independent experiments. (**F**) Effect of AHR siRNA on TF mRNA expression after a 4 h stimulation with 200µM IS. Data represent the mean ± SEM of 5 independent experiments. *p < 0.05, **p < 0.01.

Laminar shear stress (Figure 5A) and IS stimulation (Figure 5B) increased mRNA expression of TF in a time dependent manner. Under shear stress conditions, TF expression was increased at 4 h (F3 mRNA fold change at 4 h: 4.7 ± 1.2) and maximal at 24 h (F3 mRNA fold change at 24 h: 10.8 ± 2.2). TF upregulation by IS had the same kinetic profile but was lower than that induced by shear stress (TF mRNA fold change: 3.1 ± 0.3 at 4 h; 4.3 ± 0.9 at 24 h), whatever the time (Figure 5B). In endothelial cells stimulated with IS under shear stress conditions, IS amplified shear stress-induced mRNA expression of TF (Figure 5F). Shear stress and IS stimulation also increased TF protein expression at 4 h in a similar way (Figure 5C). Again, IS amplified shear stress-induced protein expression of TF (Figure 5C).

We verified that AHR did not bind to *F3* promoter to induce TF expression in HUVEC stimulated with IS. ChIP experiments demonstrated no enrichment of AHR on *F3* promoter, showing IS stimulation did not induce AHR recruitment to *F3* promoter (Figure 5D). As expected, AHR was recruited to *CYP1B1* promoter, which is known to contain XRE sequences, after HUVEC stimulation by IS (Figure 5D). 

We finally analyzed the effect of AHR inhibition in TF induction by shear stress and IS. AHR siRNA and AHR inhibitor CH223191 significantly suppressed shear stress-induced TF expression (Figure 5E). In addition, AHR inhibition by AHR siRNA significantly decreased indoxyl sulfate-induced TF upregulation under static and under shear stress conditions (Figure 5F). 

### 2.5. Genistein Suppressed Shear Stress-Mediated but not Indoxyl Sulfate-Mediated TF Expression

**Figure 6:**
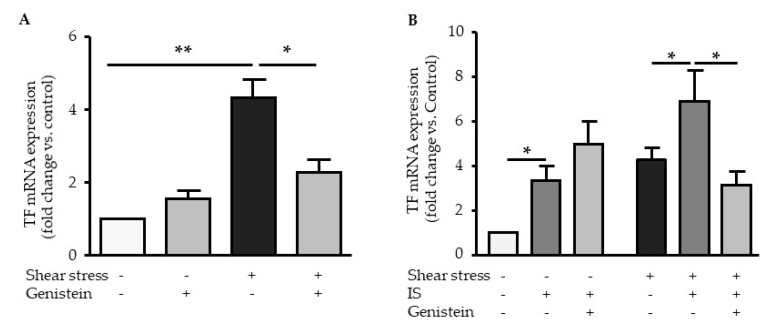
Effect of genistein on TF induction by shear stress and IS. (**A**) Effect of genistein (100 µM) on mRNA expression of TF after a 4 h of shear stress (5 dynes/cm^2^). (**B**) Effect of genistein (100 µM) on mRNA expression of TF after a 4 h stimulation with IS (200 µM) under static or shear stress (flow) conditions. Data, expressed as mRNA fold change vs. control, represent the mean ± SEM of 5 independent experiments. *p < 0.05, **p < 0.01.

We studied the effect of genistein on shear stress- and IS-mediated induction of TF mRNA. Genistein significantly decreased shear stress-induced TF mRNA expression (Figure 6A) but did not inhibit IS-induced TF mRNA expression under static conditions (Figure 6B). In HUVEC stimulated with IS under shear stress conditions, genistein partly inhibited TF expression (Figure 6B). TF mRNA expression in HUVEC stimulated with IS under shear stress and genistein conditions was not different from TF expression in HUVEC stimulated with IS under static conditions without genistein (Figure 6B). This suggests that in HUVEC stimulated with IS under shear stress, genistein inhibits the shear stress-mediated induction of TF rather than the IS-mediated (Figure 6B). 

### 2.6. Indoxyl Sulfate Increases the Procoagulant Activity of TF under Fluid Shear Stress

To determine whether increased TF production induced by shear stress and IS was functionally active for coagulation, we measured TF-dependent procoagulant activity in HUVEC by analyzing factor Xa generation. 

**Figure 7:**
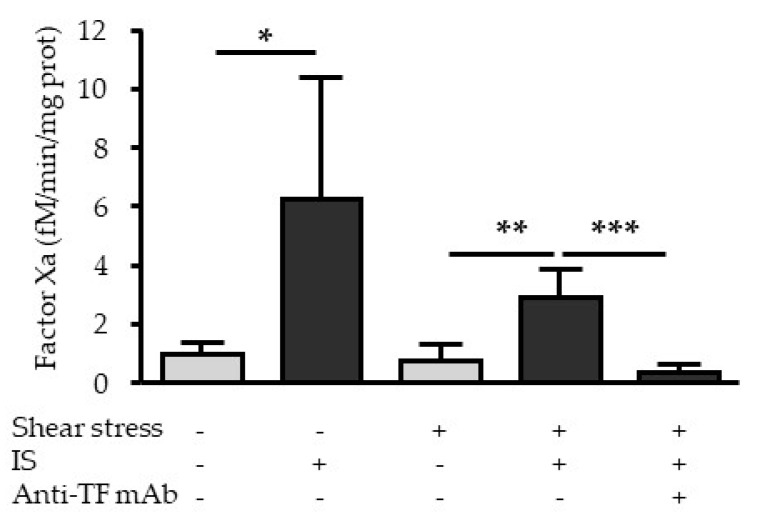
Effect of shear stress and IS on TF procoagulant activity. HUVEC were subjected to shear stress (5 dynes/cm2) and/or IS (200 µM) during 6 h, and TF-dependent procoagulant activity was studied by measuring the generation of factor Xa. A blocking TF antibody was used to confirm that factor Xa generation was dependent on TF. Data, expressed in fM/min/mg protein of factor Xa, represent the mean ± SEM of *n* = 7 independent experiments. *p < 0.05, **p < 0.01, ***p < 0.001.

Shear stress (Figure 7) did not increase TF procoagulant activity in HUVEC (factor Xa generation: 0.96 ± 0.42 fM Xa/mg protein in control cells vs. 0.77 ± 0.56 fM Xa/mg protein in cells subjected to shear stress). Under static conditions, IS increased factor Xa generation from 0.96 ± 0.42 fM Xa/mg protein in control cells to 6.28 ± fM Xa/mg protein in cells stimulated with IS (Figure 7). Under shear stress conditions, IS still increased factor Xa generation to 2.93 ± 0.97 fM Xa/mg protein, but this increase was smaller than under static conditions (Figure 7). A blocking TF antibody abolished factor Xa generation (Figure 7), confirming that factor Xa generation is related to TF activity.

## 3. Discussion

In the present study, we compared AHR activation by a physiological mechanical factor and by an endogenous toxic ligand, that is, shear stress and IS, respectively, and analyze the consequences on TF upregulation.

We first investigated AHR and AHRR expression after shear stress and IS treatment. AHRR is a bHLH-PAS protein that inhibits both induced and constitutive AHR transcriptional activity by competing with AHR for ARNT [8]. In a negative feedback loop, AHR induces the transcriptional upregulation of AHRR, which subsequently mediates suppression of AHR activity [8]. *AHRR* contains putative XRE binding sites for AHR, and close binding sites for SP1, SP3 and NF-κB transcription factors [8]. Using small interfering RNA knockdown of AHR and pharmacological AHR inhibition with CH223191, we demonstrated the involvement of AHR in shear stress- and IS-induced AHRR upregulation, which was very dependent on AHR. AHR activation by exogenous AHR ligands generally increase the mRNA levels of AHRR, which, in turn, affects the induction of AHR target genes and regulates the response to AHR agonists [8]. This model of AHR/AHRR feedback loop could also apply to “physiologically” activated AHR, like endogenous ligand-activated AHR [8] or shear stress-activated AHR. Therefore, AHR/AHRR may govern tissue-specific differences in sensitivity to AHR agonists as well as the constitutive response to shear stress. We found here that shear stress and IS affect AHR and AHRR expression differently. Whereas shear stress increased AHR mRNA expression, as previously shown by Han et al. [15], IS activated AHR without increasing AHR mRNA. In addition, shear stress induced a continuous increase in AHRR expression, whereas IS induced a rapid peak of AHRR expression at 4 h, which then decreased at 24 h. Flow-mediated AHR/AHRR activation may be a physiological mechanism that aims to protect the vascular wall, which is different from pathological AHR activation induced by toxic AHR agonists. In the context of CKD, high levels of IS may induce an imbalance in AHR/AHRR that can impair physiological AHR regulation. 

To investigate the consequences of AHR activation, we analyzed the overexpression of AHR target genes *PTGS2* (encoding for COX2), *CYP1A1*, and *CYP1B1*. The upregulation of CYP1A1 and CYP1B1 in endothelial cells by shear stress and IS has been well documented [12,14,15,19,20]. *CYP1A1* and *CYP1B1* expression is highly regulated by the AHR genomic pathway through the binding of activated AHR on XRE in their promoters [7]. Depending on the cell type and AHR agonist, COX2 can be regulated via the genomic and the non-genomic pathways of AHR activation [9,10,11]. Using experiments of AHR inhibition, we demonstrated that both shear stress and IS upregulate COX2, as well as CYP1A1 and CYP1B1 mRNA expression, in an AHR-dependent way. We examined the effect of genistein treatment, which was shown to inhibit AHR nuclear translocation [18]. Genistein activities as an AHR agonist and antagonist depend on cell context [21]. Genistein, significantly, but not completely inhibited the upregulation of COX2 under basal, shear stress, and IS conditions, whereas it completely inhibited the increased expression of genes regulated by the AHR genomic pathway *CYP1A1* and *CYP1B1*. This suggests COX2 upregulation in endothelial cells could occur via both genomic and non-genomic pathways of AHR activation. 

Whereas the mechanisms of AHR activation by IS have been well studied, the mechanisms of AHR activation by shear stress are still obscure. One could hypothesize that shear stress activates AHR through a mechanotransduction-sensitive signaling pathway involving mechanotransducers such as ion channels, integrins, or G protein-coupled receptors. Another hypothesis could be that shear stress induces the production of AHR-activating factors. This hypothesis was confirmed by the study of McMillan and Bradfield, which showed that shear stress can modify low density lipoproteins (LDL) and convert them to AHR-activating species [22]. We therefore tested the effect of the pharmacological AHR inhibitor CH223191, a ligand selective AHR antagonist, which preferentially inhibits some classes of AHR agonists (like 2,3,7,8-tetrachlorodibenzo-p-dioxin and related HAHs) but not others (like flavonoids and indirubin) [23]. Our results showing that CH223191 strongly inhibited shear stress mediated CYP1A1 and CYP1B1 (and, to a lesser extent, COX2) upregulation led us to suppose that if shear stress produces an AHR agonist, this agonist is preferentially inhibited by CH223191. This does not exclude that shear stress activates AHR through mechanotransducers, and further studies are required to confirm these hypotheses. When we analyzed whether shear stress and IS have additive or antagonist effects, we observed additive an effect on COX2, CYP1A1, and CYP1B1 upregulation. This suggests the existence of a synergistic mechanism of AHR activation between IS and shear stress, in which the effect of IS is not antagonized by a shear-stress produced AHR ligand or an AHR beneficial signaling pathway. The physiological AHR activation induced by shear stress does not counter the negative effect of IS nor antagonize endothelial response to this uremic toxin. 

Elevated levels of AHR agonists in CKD patients, associated with increased cellular AHR activation of AHR are likely harmful. In vascular cells exposed to indolic uremic toxins IS and IAA, AHR activation leads to the increased expression of TF, the major initiator of coagulation [12,13,24]. We showed that AHR-mediated TF upregulation is different from the upregulation of genes from the AHR genomic pathway. With IAA, we previously demonstrated that TF upregulation does not involve AHR binding to the *F3* promoter, and occurred via a non-genomic pathway in which AHR activation by IAA leads to p38MAPK activation, followed by NF-κB nuclear translocation, and NF-κB binding to *F3* promoter [13]. Here, we confirmed that IS, like IAA, does not induce AHR binding to *F3* promoter after endothelial stimulation, supporting that TF upregulation via IS/AHR is mediated by a non-genomic pathway. Interestingly, we found that genistein, which inhibits AHR nuclear translocation [18] and the upregulation of genes from the AHR genomic pathway, does not inhibit TF mRNA upregulation mediated by IS. This is consistent with an upregulation of TF independent of AHR genomic pathway.

Here, in addition to its role in IS-mediated TF induction, we demonstrated the involvement of AHR in shear-stress-mediated TF upregulation. Our experiments demonstrated an inhibitory effect of genistein on shear stress-mediated TF induction. This suggests that the AHR-dependent mechanisms involved in TF upregulation by shear stress and by IS are different. Mechanisms related to shear stress-induced TF expression appeared to be dependent on tyrosine kinases, while those related to IS appeared to be independent. One can therefore suppose that TF induction mediated by the IS/AHR axis is different from that mediated by the shear stress/AHR axis.

There are three different forms of TF at the cell surface. In non-stimulated cells, TF is expressed in a cryptic form that is inactive [25,26]. In cells stimulated with specific agonists, TF binds the serine protease factor VIIa and is converted into a signaling form that cleaves protease-activated receptor 2 (PAR2), or into a procoagulant form that initiates coagulation via the conversion of factor X into the active procoagulant factor Xa [25,26]. Here, we focused on the transition from the inactive to the procoagulant form of TF after upregulation of TF by shear stress or IS. By measuring Factor Xa generation, we showed that the increase in TF expression induced by shear stress is not associated with an increase in its procoagulant activity. On the contrary, IS increased both expression and procoagulant activity of endothelial TF. Therefore, in the presence of IS, endothelial cells acquire a procoagulant phenotype, even under antithrombotic shear stress conditions. 

TF contains allosteric disulfide bond Cys186-Cys209, whose redox state is crucial for the shift between the different forms of TF [26]. S-nitrosylation of TF and S-nitrosoglutathione, the S-nitro derivative of glutathione, appear to maintain TF in its encrypted inactive form [26]. On the other hand, the TF procoagulant form involves the oxidation of TF allosteric disulfide bond Cys186–Cys209 that increases TF affinity for Factor VIIa and favors the recognition of Factor X [25]. To explain our results, we can assume that IS and shear stress affect different factors involved in the redox state of the endothelial cell and per se TF. The production of NO by endothelial cells under laminar shear stress [27] could maintain TF in a cryptic non-coagulant state, in agreement with the well-known atheroprotective, anticoagulant, and anti-inflammatory properties of shear stress [16]. On the other hand, by inducing a significant decrease in intracellular glutathione [28], IS may favor a procoagulant de-encrypted state of TF. IS gives a procoagulant phenotype to endothelial cells that persists even under antithrombotic shear stress conditions.

In conclusion, shear stress and IS activated AHR through different pathways, but both increased TF expression in an AHR-dependent way. Shear stress did not induce TF activity, whereas IS increased it, even under antithrombotic shear stress conditions. This supports the role of IS as a pro-thrombotic factor in CKD.

## 4. Materials and Methods

### 4.1. Endothelial Cell Culture 

HUVEC were obtained from umbilical cord vein by collagenase digestion as described previously [29] and grown to the fourth passage in EGM-2 medium (Lonza, Levallois-Perret, France) (containing 2% fetal bovine serum), under standard cell culture conditions (humidified atmosphere at 37 °C, 5% CO_2_). 

### 4.2. Incubation with the Uremic Toxin IS

Cells were incubated for indicated times in the presence of IS (Sigma-Aldrich, Saint Quentin Fallavier, France) at 200 µM, a concentration in the highest range described in uremic patients [30]. IS was diluted 1/1000e from a 200 mM stock solution. KCl (Sigma Aldrich, Saint Quentin Fallavier, France) at the same concentration was used as control because IS was supplied by the manufacturer as a potassium salt. Cells were treated with the AHR Inhibitor CH-229131 (Sigma-Aldrich) at 10 µM, with AHR siRNA (see below), or with genistein 100 µM (Calbiochem Merck, Saint Quentin Fallavier, France). CH223191 and genistein were diluted 1/1000e from stock solutions in dimethylsulfoxyde (DMSO). Cells were preincubated with CH223191 or genistein for 1 h and exposed to shear stress and/or IS in the presence of inhibitors. 

### 4.3. SiRNA Knockdown of AHR 

HUVEC were transfected with siRNA control (Negative Universal Control, Stealth™ RNAi, Life Technologies, Courtaboeuf, France), or a pool containing 3 Silencer^®^ Select siRNA directed against AHR (1200, 1999 and 1998, Life Technologies, Courtaboeuf, France) by magnetofection using SilenceMag beads (OZ Biosciences, France), according to the manufacturer’s instructions. Cells were detached with trypsin-EDTA solution 24 h post-transfection and plated at 1.5 × 10^6^ cells/mL in EGM2 medium. Forty-eight hours post-transfection, cells were subjected to static, shear stress and/or IS conditions. 

### 4.4. Flow System

HUVEC were seeded at 10^6^ cell/mL onto tissue-culture-treated microslides (Ibidi, Clinisciences, Nanterre, France) and cultured for 24 h. Cells were subjected to a laminar shear stress of 5 dynes/cm2 using the Ibidi perfusion system (Ibidi, Clinisciences, Nanterre, France) to simulate venous shear stress.

### 4.5. RNA Extraction and Quantitative RT-PCR Analysis of mRNA Expression

Total RNA was extracted by an RNeasy mini-kit (Qiagen, Courtaboeuf, France). Reverse transcription (RT) was performed on 500 ng of total RNA using the Takara PrimeScript™ RT reagent Kit (Ozyme, Saint Quentin en Yvelines, France) followed by quantitative polymerase chain reaction (qPCR) on 25 ng of cDNA using the Takara SYBR qPCR Premix Ex Taq (Ozyme, Saint Quentin en Yvelines, France). We quantified the mRNA expression of the following genes: *AHR*, *F3* (TF), *PTGS2* (COX2), *CYP1A1*, *CYP1B1*, and *AHRR*. The housekeeping gene *HPRT* was used to normalize the target gene values. The sequences of primers were as follows: AHR forward: 5‘ATACTATGCTGGGGCCATGT3′, AHR reverse: 5‘GCTCAAGTCGGACGAATAGG3′; F3 forward: 5‘TGCAGTAGCTCCAACAGTGC3′, F3 reverse: 5‘GAGTGTATGGGCCAGGAGAA3′; COX2 forward 5′CAGGATACAGCTCCACAGCA3′, COX2 reverse: 5′ATTGACCAGAGCAGGCAGAT3′; CYP1A1 forward: 5′GACAGATCCCATCTGCCCTA3′, CYP1A1 reverse: 5′ATAGCACCATCAGGGGTGAG3′; CYP1B1 forward: 5′TGATGGACGCCTTTATCCTC3′, CYP1B1 reverse: 5′CCACGACCTGATCCAATTCT3′; HPRT forward: 5′GGATTATACTGCCTGACCAAGGAAAGC3′, HPRT reverse: 5′GAGCTATTGTAATGACCAGTCAACAGG3′.

All PCR reactions were performed with the Applied Biosystems StepOnePlus Real-Time PCR System (Thermofisher Scientific, Villebon-sur-Yvette, France). The fold change of mRNA expression versus control condition was calculated using the 2^−ΔΔCt^ method. The transcript for the housekeeping gene HPRT was used for data normalization. 

### 4.6. Chromatin Immunoprecipitation (ChIP) Assay

HUVEC monolayers (6 × 10^6^ cells) were treated with IS (200 µM) or KCl (control) for 60 min. Cells were fixed by adding formaldehyde directly to the medium to a final concentration of 1% and incubated for 10 min at room temperature, then 40 min at 4 °C. ChIP assay was performed using the EZ-Magna ChIP™ A/G Chromatin Immunoprecipitation Kit (Merck Millipore, Guyancourt, France), according to the manufacturer’s instructions. Immunoprecipitations were performed overnight at 4 °C with 5 µg of rabbit IgG (control IgG) or rabbit polyclonal antibody against AHR (Santa Cruz Biotechnology, Clinisciences, Nanterre, France). Real-time PCR quantification of ChIP enrichments were run on Applied Biosystems StepOnePlus Real-Time PCR System (Thermofisher Scientific, France) using the Takara SYBR qPCR Premix Ex Taq (Ozyme, Saint Quentin en Yvelines, France). Specific primer sequences for promoters were as follows: TF forward: 5′-GCCCTCCCTTTCCTGCCATAGA-3′, TF reverse: 5′-CCTCCCGGTAGGAAACTCCG-3′; CYP1B1 forward: 5′-ATATGACTGGAGCCGACTTTCC-3′, CYP1B1 reverse: 5′-GGCGAACTTTATCGGGTTGA-3′. Fold enrichment was calculated using the 2^−ΔΔCt^ method, where ΔΔCt represents the difference between threshold cycles of experimental rabbit polyclonal antibodies against AHR over rabbit control IgG.

### 4.7. Measurement of TF by Enzyme-Linked Immunosorbent Assay

TF protein was quantified in HUVEC lysates with the Quantikine Human Coagulation Factor III/Tissue Factor (R&D Systems, Lille, France) enzyme-linked immunosorbent assay (ELISA) kit, according to the instructions of the manufacturer.

### 4.8. Procoagulant Activity of TF

Cell TF activity was studied by measuring TF capacity to generate factor Xa. Cells were lysed with lysis buffer containing Tris-HCl pH 7.5, NaCl, and 0.1% Triton X-100. Three cycles of freezing/defreezing were performed to lyse the cells while preserving TF activity. Protein concentration was measured with the Pierce™ Bicinchoninic Acid Protein Assay (ThermoFisher Scientific, Villebon-sur-Yvette, France). Samples were diluted ½ in HEPES buffer then incubated with a blocking anti-TF antibody (clone VD8, SEKISUI Diagnostics, Pfungstadt, Germany) or an irrelevant IgG1 mAb to determine the TF contribution in factor Xa generation.

Cell lysates were incubated during 2 h at 37 °C in buffer containing factor X, factor VII and calcium to allow factor Xa generation. Reaction was stopped with EDTA. A fluorescent substrate of factor Xa was added and fluorescence values (excitation 390nm/emission 460nm) were measured during 15 min at 37 °C using a fluoroskan Ascent (ThermoFisher Scientific, Villebon-sur-Yvette, France). Data were normalized with protein level, and results were expressed in fM of FXa produced per minute per mg protein. 

### 4.9. Statistical Analyses

Statistical analyses were performed with the Prism (GraphPad Software Inc, San Diego, CA, USA). Significant differences were revealed by the Wilcoxon signed rank test or by the Mann Whitney test. Data are expressed as mean ± SEM of independent experiments performed on different cell preparations. 

## Abbreviations

AIP	aryl hydrocarbon receptor interacting protein
AHR	aryl hydrocarbon receptor
AHRR	aryl hydrocarbon receptor repressor
ARNT	aryl hydrocarbon nuclear translocator
ChIP	chromatin immunoprecipitation
CKD	chronic kidney disease
COX2	cyclooxygenase-2
CYP1A1	cytochrome P450 family 1 subfamily A member 1
CYP1B1	cytochrome P450 family 1 subfamily B member 1
HSP90	heat shock protein 90kDa
HUVEC	human umbilical vein endothelial cells
IAA	indole-3 acetic acid
IS	indoxyl sulfate
MAPK	mitogen-activated protein kinases
NF-κB	nuclear factor-kappa B
qPCR	quantitative polymerase chain reaction
RT	reverse transcription
TF	tissue factor
XRE	xenobiotic response element
